# Statistical Size Effect in Fatigue Properties for Mini-Specimens

**DOI:** 10.3390/ma13102384

**Published:** 2020-05-22

**Authors:** Tomasz Tomaszewski

**Affiliations:** Faculty of Mechanical Engineering, University of Science and Technology, al. Prof. S. Kaliskiego 7, 85-796 Bydgoszcz, Poland; tomaszewski@utp.edu.pl

**Keywords:** mini-specimen, size effect, Weibull distribution, high-cycle fatigue, macro-fractography

## Abstract

The study verifies the sensitivity of selected construction materials (S235JR structural steel and 1.4301 stainless steel) to the statistical size effect. The *P–S–N* curves were determined experimentally under high-cycle fatigue conditions for two specimen sizes (mini-specimen and standard specimen). The results were analyzed using a probabilistic model of the three-parameter Weibull cumulative distribution function. The analysis included the evaluation of the technological process effects on the results based on the material microstructure near the surface layer and the macro-fractography. The differences in the susceptibility to the size effect validated the applicability of the test method to mini-specimen and showed different populations of the distribution of critical material defects.

## 1. Introduction

Structural and mechanical components under variable loads are susceptible to fatigue crack initiation and propagation. Due to the effect of multiple factors on the fatigue failure, it is necessary to determine the actual material data (unnotched specimens) based on the dedicated standards, guidelines, directives and recommendations, and the basic deterministic models and correction factors. In some cases, the analysis is difficult or impossible. Researchers suggest a newly developed test method based on non-standard specimens.

One of the research areas is specimen miniaturization. The development of procedures to identify fatigue properties, cracking resistance, and propagation of fatigue cracking of materials used in nuclear reactors (small specimen test technique) is aimed at the use of mini-specimens with reduced volume due to limited irradiation volume and material availability [1,2,3]. The specimen miniaturization is required to test the local fatigue strength, allowing for the morphology of thin-walled material grains [4,5], different welded joint areas (seam, heat-affected zone, base metal) [6], limited microstructural properties of the material [7], or the reduction in fatigue test costs [8]. The developed test method involves the tests in the gigacycle fatigue range determined using non-standard test stands and an ultrasound technique (20 kHz frequency). The technical capabilities of the fatigue tests (reduced load range) and the required cooling of the specimens (improved heat transfer) require small-diameter specimens [9].

Any change in specimen size affects its mechanical and fatigue properties. Failure to allow for the size effect may introduce significant errors in the evaluation of material and structural element properties. Determining this effect, i.e., extrapolating the test results to specimens with different dimensions, is a significant factor in the engineering practice, and new analysis methods are constantly being developed [10,11,12].

The size effect results from the scaling process inaccuracy, i.e., change in dimensions of the selected specimens by a constant coefficient. There are no methods available to scale all the product properties at a constant level (e.g., material structure, changes in surface layer or specimen surface). The specimen size is changed by maintaining the dimensional ratio of the specimen. The scaling procedures and rules are specified in the probability theory, which allows carrying out the experimental tests on a reduced size model and easily converting the results to a different scale. One of the main assumptions is the proportional change of dimensions into values that are significant for the analyzed process [13].

It is widely accepted that the material strength depends on the specimen size for monotonic or fatigue loads. Most authors of experimental studies confirmed that smaller specimens have higher strength, which is definitely true in the macro scale. As the specimen size approaches the grain size, other failure mechanisms can be observed (dislocation slip, plastic strain gradient) that introduce discrepancies between the results for different scale ranges. The scope of presented tests includes the macro scale, where the change in strength results from the effect of categorized factors due to the random distribution of material defects (statistical size effect), shape, and load type (geometric size effect), as well as the effect of technological processes used in element manufacturing (technological size effect) [14].

The presented study describes the statistical size effect for two construction materials (S235JR structural steel, 1.4301 stainless steel) showing different sensitivity to the size effect. High-cycle fatigue tests were carried out under axial loading conditions for a mini-specimen and a standard specimen. The study aimed to determine whether the statistical size effect is visible in fatigue properties in relation to the analysis of the grain size, as well as the crack propagation based on the macro-fractography and *P–S–N* curves.

## 2. Materials and Methods

### 2.1. Statistical Size Effect

**Figure 1:**
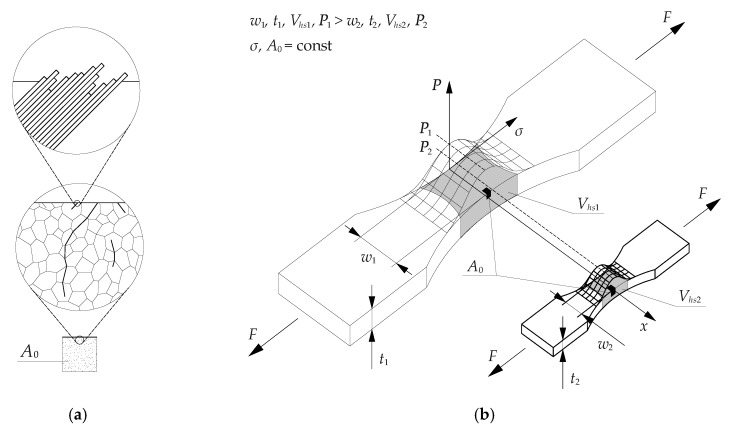
Statistical size effect: (**a**) mechanism of fatigue crack initiation; (**b**) probability of crack initiation.

The material defects (point defects, e.g., small pores, inclusions; linear defects, e.g., dislocation, microcracks; surface defects, e.g., grain boundaries) in the reference size *A*_0_ are randomly distributed in any specimen size. The defects cause fatigue crack initiation from the local plastic strains in the form of slip bands that further propagate with an increase in the number of cycles to form clusters and bundles. The cracks initiate due to local stress concentration (Figure 1a). Local stress determines the fatigue strength. The crack initiation depends on the material condition, load, and number of cycles.

In the reference size *A*_0_, the cracks propagate independently. Figure 1b shows the comparison of the stress *σ*(*x*, *y*, *z*) in two specimens. The stress distribution depends on the geometry and specimen size and is not affected by the load level applied. A higher probability of crack initiation will be observed in the larger specimen due to the higher number of statistically distributed defects (statistical size effect). The probability of identifying a critical defect increases with the increase of highly stressed volume (*V_hs_*).

The statistical size effect is based on Weibull’s weakest-link theory model [15], which describes the scatter of material strength properties. This approach is used to qualitatively determine the effect of specimen size on its fatigue strength. A standard form of the Weibull distribution for the failure probability is

(1) $$\;P\left(\sigma ,\;N\right)=1-\exp\left[-\frac{1}{A_{0}}\;\overset{\;}{\underset{A}{\int }}f\left(\sigma ,\;N\right)dA\right],$$

where *A*_0_ is the reference size (length, area, volume), and *f*(*σ, N*) is the failure probability function.

The probability of failure for different defect distributions can be described by the three-parameter Weibull cumulative distribution function. For the distribution of fatigue strength of an element with the same geometry and stress distribution, the function takes the form

(2) $$P\left(\sigma ,\;N\right)=1-\exp\left[-\frac{A}{A_{0}}\;{\left(\frac{(\sigma ,\;N)-\lambda }{\delta }\right)}^{\alpha }\right],$$

where *α* is the shape parameter, *σ* is the applied stress, *N* is the corresponding cyclic lifetime, *λ* is the location parameter, and *δ* is the scale parameter. After transforming Equation (2), we get the form

(3) $$P\left(\sigma ,\;N\right)=1-\exp\left[-{\left(\frac{(\sigma ,\;N)-\lambda }{\delta^{*}}\right)}^{\alpha }\right],$$

and

(4) $$\delta^{*}={\left(\frac{A_{0}}{A}\right)}^{\frac{1}{\alpha }}.$$

If the experimental test results are defined with the expansion of Equation (2), the results can be correlated with the results for specimens with different cross-sectional areas. The location parameter λ, obviously contrary to the scale parameter *δ*, does not depend on the size effect. The shape parameter *α* depends on the stress and the function of the defect in the reference size *A*_0_. According to Equation (4), it is possible to propose a statistical size coefficient. This coefficient can be determined for uniaxial stress condition, identical failure probability, and two different specimen sizes.

(5) $$n_{s}=\frac{\sigma_{2}}{\sigma_{1}}={\left(\frac{A_{1}}{A_{2}}\right)}^{\frac{1}{\alpha }},$$

where *σ*_1_ is the estimated fatigue strength for a specimen with determined cross-sectional area *A*_1_, and *σ*_2_ is the fatigue strength for a specimen with known cross-sectional area *A*_2_. The shape parameter α can be estimated based on the fatigue strength of two specimen sizes. The equation shows that the 1/*α* exponent is a significant factor in the size effect and indicates that a scatter of data depending on the reference size must be allowed for in the analysis. This explanation is based on the interpretation of the applied stress, the strength of the element, and the probability of coming across a critical material defect [16,17].

### 2.2. Material and Chemical Composition

The analysis of the size effect was carried out for S235JR structural steel and 1.4301 (304) stainless steel—two materials commonly used in machine design due to combined good mechanical properties, machinability, availability, and price. Both materials show different sensitivity of mechanical properties to changes in cross-sectional area. The 1.4301 steel has a significantly higher content of alloying elements compared to other commonly used steels, including S235JR. Table 1 and Table 2 show the chemical compositions of the steels. For 1.4301 steel, the high corrosion resistance is due to the chromium content of 18.1%. A nickel content of 8.1% mainly stabilizes the austenite to room temperature.

**Figure 2:**
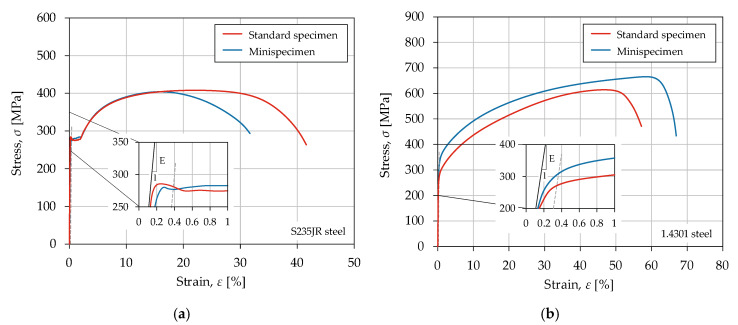
Stress–strain curve for steels: (**a**) S235JR; (**b**) 1.4301.

The mechanical tests were performed on an Instron 8874 material testing machine (Instron Worldwide Headquarters, Norwood, MA, USA). The tests were carried out using a ±25 kN forced gauge and 2620 Instron Dynamic Extensometer (Instron Worldwide Headquarters, Norwood, MA, USA) with a basic gauge length of 25 mm and ±5 mm extension. The static tensile test was carried out in accordance with PN-EN ISO 6892-1:2016 [20] for unnotched specimens. Two sizes of unnotched specimens were analyzed. The minimum cross-sectional areas of the mini-specimen and the standard specimen were 3.5 and 28 mm^2^, respectively. Figure 2 shows the quasi-static characteristic (*ε-σ* curve). Table 3 shows the average values of the mechanical properties.

The mechanical properties of S235JR steel are independent of the specimen size. An increase in tensile strength and yield strength was observed for 1.4301 steel compared to the standard specimen. The mini-specimen showed higher values for both properties. The tensile strength ratio of the mini-specimen to the standard specimen was equal to 1.07. In stainless steel, the deformation is carried out by plastic slip, twinning, or martensitic transformation. The size effect occurs when there are insufficient dislocation sources available in the volume tested such that large stresses are required to initiate plasticity, followed by continued plasticity at much lower stresses [21].

**Figure 3:**
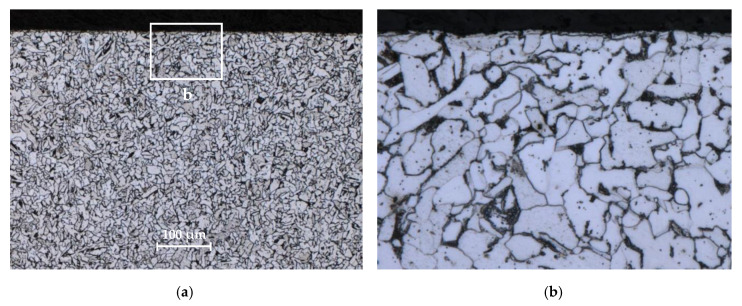
Microstructure of S235JR steel: (**a**) general view; (**b**) enlarged marked area.

**Figure 4:**
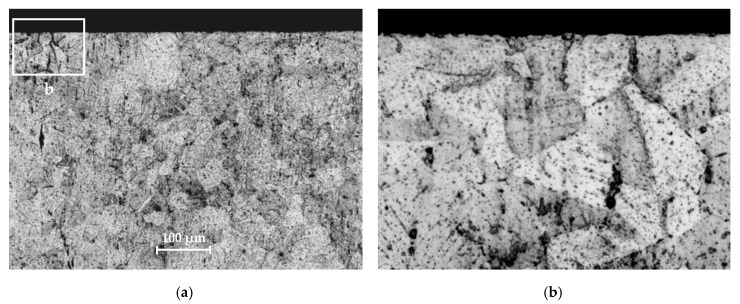
Microstructure of 1.4301 steel: (**a**) general view; (**b**) enlarged marked area.

Figure 3 and Figure 4 show the microstructure of tested materials for mini-specimens. For S235JR steel, light ferrite areas and dark fine perlite areas are visible. Austenite grains are visible in 1.4301 steel. The material microstructure was evaluated using an OLYMPUS LEXT OLS4100 confocal laser scanning microscope (Olympus, Tokyo, Japan). Prior to the structural analysis, the specimens were subjected to metallographic preparation, involving grinding on SiC abrasive papers (240 to 2400 grade) and polishing with 3-μm and 1-μm diamond suspensions.

The analysis of grain size *d* involved a comparison of the experimental values of logarithmic normal distribution. Grain sizes were measured for each of the tested materials and specimen sizes. The measurement was taken in the center of the specimen at a distance of 500 µm from the edge of the specimen. The static distribution equation was expressed as follows:

(6) $$f\left(d\right)=\frac{1}{dk\sqrt{2\pi }}\times e^{-\frac{1}{2}{\left(\frac{\ln\left(d\right)-µ}{k}\right)}^{2}},$$

where *λ, k* are the logarithmic normal distribution parameters (size, shape).

Table 4 shows the logarithmic normal distribution parameters for grain size *d*. Similar values of average grain size (eighth column) were obtained for a given material. The sixth and seventh columns indicate the results of the statistical test *χ^2^* in accordance with the following formula:

(7) $$\chi^{2}=\overset{r}{\underset{i=1}{\sum }}\frac{{\left(n_{i}-np_{i}\right)}^{2}}{np_{i}},$$

where *r* is the number of classes, *n_i_* represents the empirical numbers of subsequent classes, *p_i_* represents the theoretical frequencies of the class, and *n* is the sample size.

**Figure 5:**
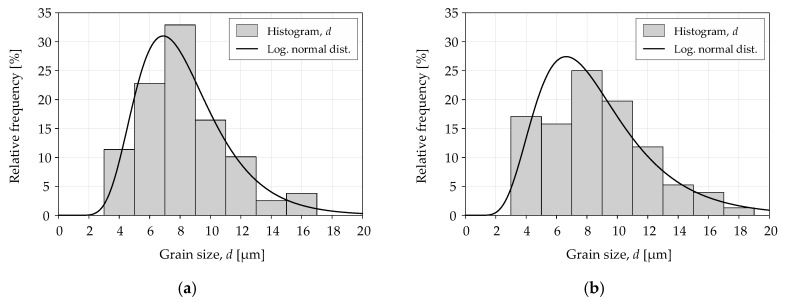
Distribution of grain size *d* for S235JR steel with the following grain locations: (**a**) center; (**b**) 500 µm.

**Figure 6:**
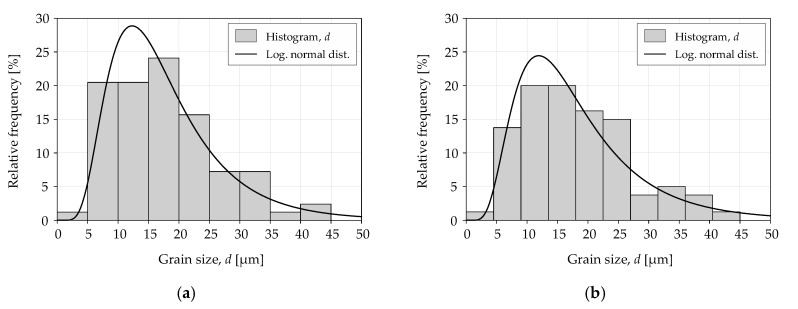
Distribution of grain size *d* for 1.4301 steel with the following grain locations: (**a**) center; (**b**) 500 µm.

Figure 5 and Figure 6 show the grain size distribution for the tested materials. The logarithmic normal distribution values were similar for different locations of a grain size measurement, which may indicate a similar distribution. The Mann–Whitney U test was conducted for two populations to verify the null hypothesis on the uniform distribution in both locations. The calculated *p*-values for S235JR steel and 1.4301 steel were 0.81 and 0.71, respectively. There was no basis to reject the null hypothesis because the calculated *p*-values were higher than the significance level of 0.05.

The grain size analysis showed no grain deformation and no size reduction in the machining area. The grains are cut, and the surface is typical for the steel machining method used. The conclusions apply to both analyzed specimen sizes.

### 2.3. Experimental Tests

**Figure 7:**
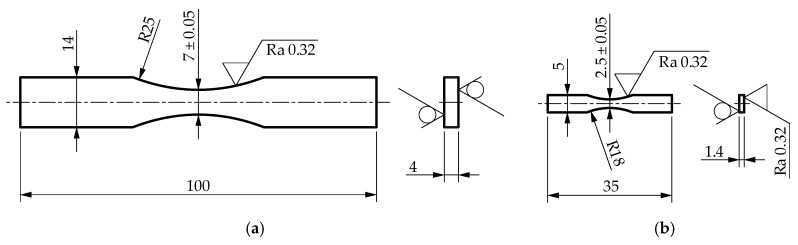
Geometry of the flat specimen for fatigue testing, cut from a 4-mm-thick plate: (**a**) standard specimen; (**b**) mini-specimen.

The fatigue property testing programs for the selected steels were carried out for two specimen sizes. The standard specimen with a cross-sectional area (28 mm^2^) corresponded to the material fatigue standards [22,23]. The cross-sectional area of the smaller specimen (3.5 mm^2^) was determined to maintain the ratio of the cross-sectional area of the standard specimen. The mini-specimen was milled in thickness. The flat sides of the standard specimen were not machined. Both specimens showed a constant theoretical stress concentration coefficient *α_k_*. Due care was taken to prepare the specimens and maintain constant parameters of the technological process. The details of mini-specimen test methods are presented in Reference [24]. Figure 7 shows the geometry and dimensions of the specimens.

The tests were carried out under high-cycle fatigue conditions for the axial load in a symmetrical sine-wave cycle (*R* = −1) in accordance with the international standards [23,25]. The purpose was to determine the *P–S–N* curve for the load-controlled test. The end criterion was the macro-cracking of the specimen. A minimum number of points for the preliminary tests was seven [26].

The fatigue characteristics are presented by approximating experimental data to a linear equation, which corresponds to a 50% failure probability. The Basquin model is used to describe the stress range and number of cycles on a log–log scale along the *S–N* field.

(8) $$N=\frac{C}{S_{ai}^{\beta }},$$

where *N* is the number of cycles to failure, *S_ai_* is the stress level, subscript *i* is the ordinal number, *C* is the constant parameter, and *β* is the slope coefficient. The spread of fatigue strength in the high-cycle range decreased with increasing stress amplitude, which did not describe the Basquin model with normal distribution [27]. The Basquin model presents undeniable contradictions, as implied by parallel percentile curves along with further evidence of the inconsistency of this model when applied to experimental programs [28,29].

The interpretation of the size effect refers to the particular *P–S–N* field model chosen. The test results were approximated using a probabilistic model based on the three-parameter Weibull cumulative distribution function for the selected failure probability. Assuming that the Weibull distribution describes the fatigue characteristic in the high-cycle fatigue range, Equation (3) is related to stress levels corresponding to the *S–N* curve. The location parameter (*λ*(*S_ai_*) = 10 *^n^*^⋅log(*S*^*_ai_*^)+*d*^) and the scale parameter (*δ*(*^S^_ai_*) = 10 *^m^*^⋅log(*S*^*_ai_*^) + *b*^) can be defined from the regression line. The parameters *n, d, m*, and *b* are the coefficients in the *S–N* curve equation. The equation for the *P–S–N* curve is defined by the following formula:

(9) $$P\left(N\right)=1-\exp\left[-{\left(\frac{\left(N_{i}\right)-\lambda \left(S_{ai}\right)}{\delta \left(S_{ai}\right)}\right)}^{\alpha }\right],$$

where *N_i_* is the number of cycles, *S_ai_* is the stress level, and subscript *i* is the ordinal number.

## 3. Results

### 3.1. Fatigue Analysis

**Figure 8:**
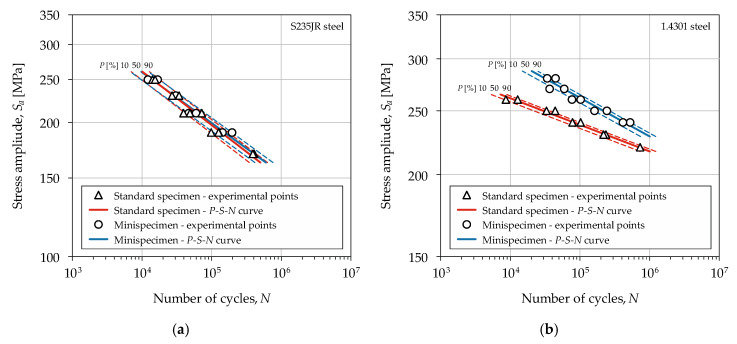
*P–S–N* curves for steel: (**a**) S235JR; (**b**) 1.4301.

Figure 8 shows the *P–S–N* curves for the high-cycle fatigue range represented by the Basquin model (Equation (8)) and the Weibull distribution (Equation (9)) for 10%, 90% failure probability. The Basquin model parameters are summarized in Table 5. The values of the Weibull distribution scale parameter *α* calculated for S235JR steel and 1.4301 steel were equal to 5.14 and 6.16, respectively. The tested materials were selected correctly since they show different sensitivity to changes in cross-sectional area. The differences in strength can be observed in the results obtained using identical procedures for specimen preparation and test conditions. A statistical size coefficient *n_s_* was calculated for the experimental values of the fatigue strength. The results were highly consistent for S235JR steel with a coefficient *n_s_* close to 1. For 1.4301 steel, the results differed with a clearly observable size effect indicated by the high *n_s_* coefficient (1.09). The fatigue strength increases with the decrease in cross-sectional area, validating the theoretical assumptions. The relationship between the *P–S–N* curves was determined statistically using a parallelism test. The test showed that, within the range of analyzed fatigue life, the *P–S–N* curves for S235JR steel are parallel.

**Figure 9:**
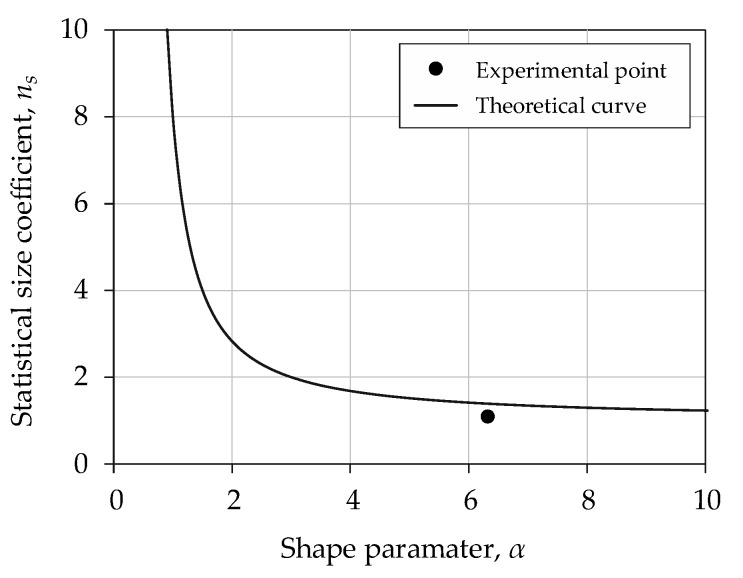
Coefficient *n_s_* to shape parameter *α* for 1.4301 steel.

The Weibull distribution shape parameter α was analyzed for two specimen sizes made of 1.4301 steel. The value of the coefficient *n_s_* was determined for high-stress volume (stress exceeds 0.95 *S_max_*). Figure 9 shows the relationship between statistical size coefficient *n_s_* and the shape parameter *α*. The marked experimental point was determined for the ratio of fatigue strength of two specimen sizes according to Equation (5). The theoretical curve was calculated from the right side of Equation (5), taking into account the shape parameter (α = 6.16). The percentage error of the statistical size coefficient *n_s_* is equal to 22.2%.

### 3.2. Macro-Fractography

**Figure 10:**
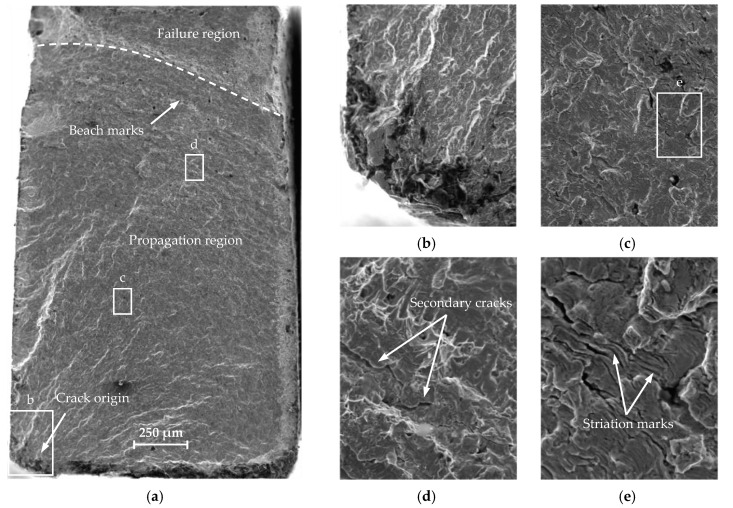
Fracture surfaces of mini-specimen made of S235JR steel: (**a**) overall surface; (**b**) crack origin; (**c**) propagation region; (**d**) secondary cracks; (**e**) striation marks.

**Figure 11:**
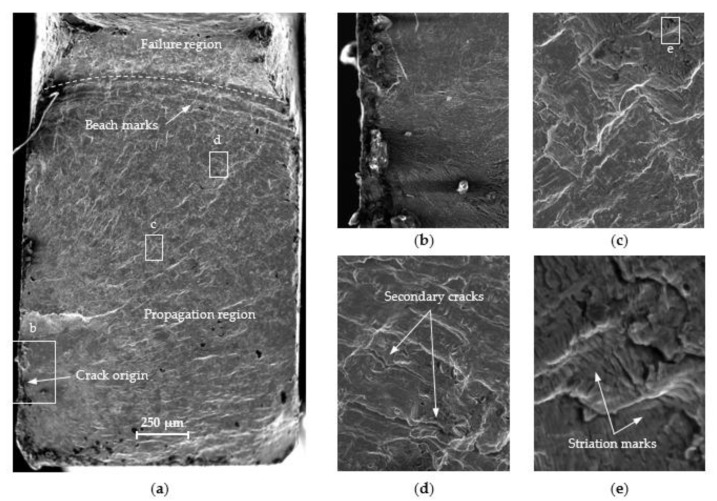
Fracture surfaces of mini-specimen made of 1.4301 steel: (**a**) overall surface; (**b**) crack origin; (**c**) propagation region; (**d**) secondary cracks; (**e**) striation marks.

**Figure 12:**
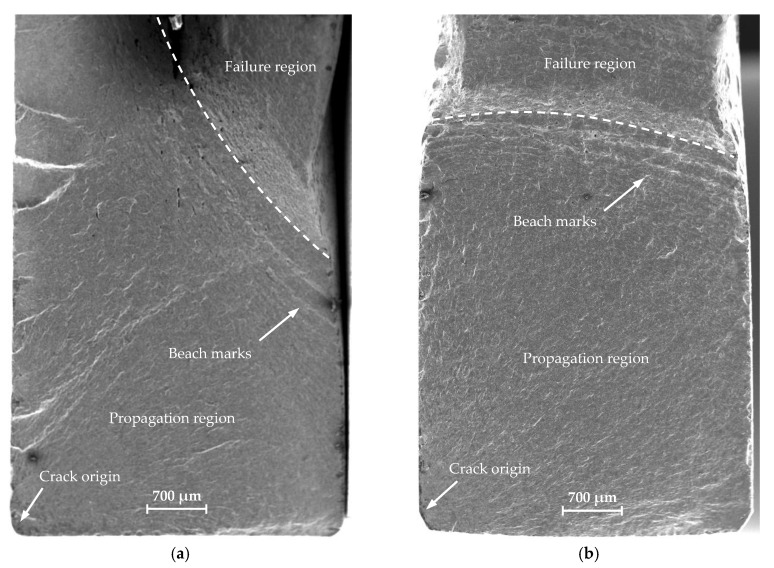
Overall fracture surfaces of standard specimen: (**a**) S235JR steel; (**b**) 1.4301 steel.

The fatigue crack propagation was analyzed based on the macro-fractography of the tested specimens. The cracks were analyzed using a JEOL JSM-6610 electron scanning microscope (JEOL, Tokyo, Japan) in a secondary electron mode (SE). The observations were performed at 15 kV acceleration voltage. The fracture surfaces were compared between the standard specimen and mini-specimen made of the same material. Figure 10, Figure 11 and Figure 12 show the sample SEM images of fractured specimens.

**Figure 13:**
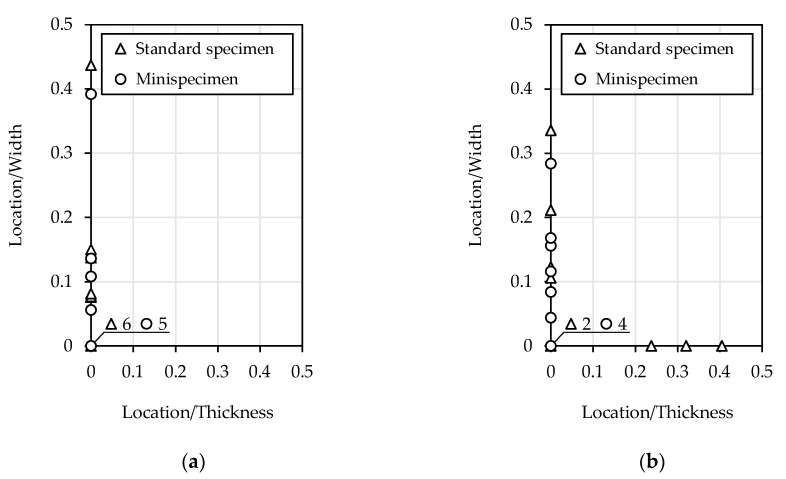
Location of crack origin for: (**a**) S235JR steel; (**b**) 1.4301 steel.

The fracture surfaces of all specimens subject to similar load amplitude show three distinct areas: a crack origin, a propagation region, and a failure region (Figure 10a, Figure 11a, and Figure 12). A crack initiation directly at or close to the specimen corner can be observed. The specimen edges were chamfered to reduce stress concentration. The cracks initiated on the specimen surface from a distinct point at which the local plastic strain occurs (Figure 10b and Figure 11b). Figure 13 shows a summary of the crack origin for all tested specimens. The region around the crack origin is of fine-grained appearance and a small roughness. This results from the crack propagation velocity and the friction of the surface of the cracks. The crack spreads as the number of cycles increases, forming a propagation region (Figure 10c and Figure 11c). The propagation region increases with the decrease in load amplitude, implying the increase in the main fracture length and increase in fatigue life. The crack propagates between two surfaces forming a small constraint area. The specimen fractures suddenly when it is not able to withstand the load, and a failure region is formed. The beach marks are visible in front of the failure region, which indicates the fatigue crack growth rate. Secondary cracks can also be observed (Figure 10d and Figure 11d). Fine undissolved particles and inclusions can inhibit crack propagation and cause striation on the fracture surface. At higher load amplitudes, the particles and inclusions leave deeper striation on the surface (Figure 10e and Figure 11e). As the load amplitude decreases, the striation depth also decreases. Similar fracture morphology was discussed in References [30,31,32]. No significant changes in the surface area of the fracture for both analyzed specimen geometries were observed. In all cases, the same features, characteristic of the fatigue failure, were observed.

The surface defects caused fatigue crack initiation. Figure 13 shows the locations of crack origin for all specimen sizes and materials. The data are the ratio of the shortest measured distance from the corner to the width of the crack initiation from surface. The number of specimen failures in the corner is shown on the bottom left side of the graph. The crack initiation on the machined flat surface for the mini-specimen was not observed. The average location of the crack origin was calculated. For S235JR steel, similar values were obtained for both specimen sizes (standard specimen—0.076, mini-specimen—0.079). The value for the standard specimen made of 1.4301 steel was higher (0.193) than that of the mini-specimen (0.082). The crack initiation was also found on the thickness of the specimen.

## 4. Discussion and Conclusions

As the specimen size decreases, there is a high probability that the material test results will be distorted by the error due to the effects of the technological process used. The reason for the technological size effect is the lack of microstructure scalability in relation to the changes in macroscopic quantities. Determining the material properties does not only relate to the metallurgical microstructure (grain size) but also to its micro-geometry (surface roughness and residual stresses). The mini-specimen size was chosen to reduce the effect of the preparation method used on the results.

The mini-specimen and standard specimen tests were carried out to verify the statistical size effect in high-cycle fatigue. Experimental data were approximated using the Basquin model and the Weibull distribution for 10%, 90% failure probability. Based on the *P–S–N* curves, S235JR steel is not sensitive to the specimen size, which is indicated by the value close to 1. The coincidence of the results indicates the correct test method and parameters of the specimen preparation procedure. The grain size analysis showed no size reduction in the machining area. The results for 1.4301 steel show the differences in fatigue properties. The coefficient *n_s_* (1.09) was compared with the tensile strength ratio (1.07). The values are similar for both metals. The discrepancies obtained for stainless steel are due to micromechanical damage (plastic slip, twinning, martensitic transformation).

The statistical size coefficient *n_s_* can be calculated analytically for a different size than determined experimentally. The assumption is a linear change in the fatigue properties depending on the specimen size. The theoretical value of the coefficient *n_s_* (1.40) was compared with the experimental value (1.09). The percentage error for steel 1.4301 is equal to 22.2%. The fatigue life estimated for the theoretical relationship estimates values below the experimental value.

The analysis of the fractography images shows that the crack propagation was similar in different size specimens. All fractures showed the characteristic features of a fatigue crack (crack origin, propagation region, beach marks, secondary cracks, and striation marks) that were independent of specimen size. The size of the analyzed cross-sectional area was sufficient since no other failure modes and crack propagation mechanisms were observed.

The surface defects in all tested specimens caused fatigue crack initiation. The crack initiates from a distinct point at which local plastic strain occurs due to the hardening and the shape of the cross-sectional area. For a rectangular cross-section of the specimen, local stress concentration in the corner should initiate a crack. If it is in a different location, the surface defect is so significant that crack origin occurs faster than in the expected location (specimen corner). This was observed in a standard specimen made of 1.4301 steel. The average distance from the corner of the crack origin is higher than other specimen sizes and S235JR steel. The standard specimen made of 1.4301 steel has more statistically significant surface defects. The statistical size effect is observed based on the higher probability of crack initiation. This effect reduced the fatigue strength of the standard specimen compared to the mini-specimen. This is in line with experimental data.

To further develop the conclusions, fatigue tests on specimens with a smaller cross-sectional area can be carried out to determine any change in the mechanism of specimen failure (crack initiation, crack propagation) or any change in the location of crack initiation depending on the specimen size.

## Figures and Tables

**Table 1:** Chemical composition of S235JR steel [18].

C %	Mn %	P %	S %	Cu %	N %
0.17	1.4	0.04	0.04	0.55	0.01

**Table 2:** Chemical composition of 1.4301 steel [19].

C %	Si %	Mn %	P %	S %	Cr %	Ni %	N %
0.02	0.41	1.54	0.028	0.001	18.1	8.1	0.051

**Table 3:** Mechanical properties.

Material	Type of Geometry	Modulus of Elasticity	Tensile Strength	Yield Strength
		*E,* MPa	*R_m_*, MPa	*R_e_*, MPa
S235JR	Standard specimen	206,217	407	278
	Mini-specimen	205,793	409	276
1.4301	Standard specimen	202,205	623	269
	Mini-specimen	203,486	667	322

**Table 4:** Values of logarithmic normal distribution parameters for the grain size *d*.

Material	Location	Parameter *µ*	Parameter *k*	Number of Grains	Statistics Test *χ*^2^	Critical Value of Stat. *χ*^2^*_kr_*	Arithmetic Average	Median	Dominant
S235JR	Center	2.053	0.351	79	1.75	7.815	8.27	7.89	9.01
	500 µm	2.069	0.421	76	4.16	7.815	8.50	7.97	3.93
1.4301	Center	2.756	0.497	83	2.76	7.815	17.70	16.21	15.12
	500 µm	2.763	0.535	80	3.23	7.815	17.98	17.32	23.01

**Table 5:** Values of the Basquin model.

Material	Type of Geometry	Constant Parameter *C*	Slope Coefficient *β*
S235JR	Standard specimen	3.57 × 10^24^	8.53
	Mini-specimen	7.24 × 10^25^	9.08
1.4301	Standard specimen	2.78 × 10^64^	25.00
	Mini-specimen	1.87 × 10^48^	17.90
